# Quantitative analysis of two COVID-19 antiviral agents, favipiravir and remdesivir, in spiked human plasma using spectrophotometric methods; greenness evaluation

**DOI:** 10.1186/s13065-023-00967-6

**Published:** 2023-06-16

**Authors:** Afnan S. Batubara, Ahmed H. Abdelazim, Ahmed A. Almrasy, Mohammed Gamal, Sherif Ramzy

**Affiliations:** 1 Department of Pharmaceutical Chemistry, College of Pharmacy, Umm Al-Qura University, Makkah, 21955 Saudi Arabia; 2 Pharmaceutical Analytical Chemistry Department, Faculty of Pharmacy, Al-Azhar University, Cairo, 11751 Egypt; 3 Pharmaceutical Analytical Chemistry Department, Faculty of Pharmacy, Beni-Suef University, Beni-Suef, 62514 Egypt

**Keywords:** Favipiravir, Remdesivir, Ratio difference, First derivative of ratio spectra, Green chemistry

## Abstract

Favipiravir and remdesivir have been included in the COVID-19 treatment guidelines panel of several countries. The main objective of the current work is to develop the first validated green spectrophotometric methods for the determination of favipiravir and remdesivir in spiked human plasma. The UV absorption spectra of favipiravir and remdesivir have shown some overlap, making simultaneous determination difficult. Due to the considerable overlap, two ratio spectra manipulating spectrophotometric methods, namely, ratio difference and the first derivative of ratio spectra, enabled the determination of favipiravir and remdesivir in their pure forms and spiked plasma. The ratio spectra of favipiravir and remdesivir were derived by dividing the spectra of each drug by the suitable spectrum of another drug as a divisor to get the ratio spectra. Favipiravir was determined by calculating the difference between 222 and 256 nm of the derived ratio spectra, while calculating the difference between 247 and 271 nm of the derived ratio spectra enabled the determination of remdesivir. Moreover, the ratio spectra of every drug were transformed to the first order derivative using ∆λ = 4 and a scaling factor of 100. The first-order derivative amplitude values at 228 and 251.20 nm enabled the determination of favipiravir and remdesivir, respectively. Regarding the pharmacokinetic profile of favipiravir (C_max_ 4.43 µg/mL) and remdesivir (C_max_ 3027 ng/mL), the proposed methods have been successfully applied to the spectrophotometric determination of favipiravir and remdesivir in plasma matrix. Additionally, the greenness of the described methods was evaluated using three metrics systems: the national environmental method index, the analytical eco-scale, and the analytical greenness metric. The results demonstrated that the described models were in accordance with the environmental characteristics.

## Introduction

**Fig. 1:**
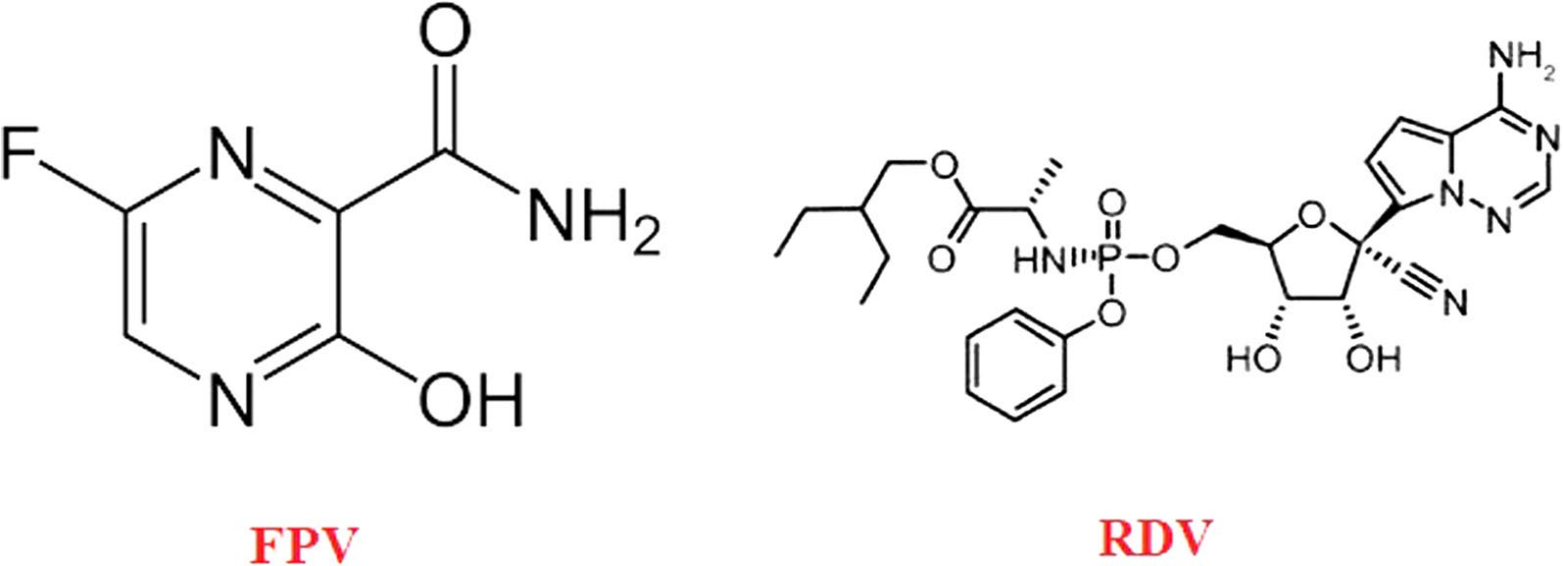
Chemical structure of FPV and RDV

 Drug repurposing has become an attractive approach to rapidly identifying drugs that combat the spread of new infectious diseases. The emergence of the COVID-19 pandemic caused by SARS-CoV-2 has prompted the search for numerous compounds that can be reused to rapidly prevent mortality, morbidity, and the spread of this novel viral disease. Despite the rapid development of COVID-19 vaccines, their proven efficacy, and massive global vaccination campaigns, it is crucial to identify different therapeutics, including effective antivirals, to address the challenges associated with failure of immunity, rejection of vaccines, and the emergence of new SARS-CoV-2 variants [1–5]. Favipiravir (FPV) and remdesivir (RDV), shown in Fig. 1, are both broad-spectrum RNA polymerase inhibitors that were first approved to treat influenza and Ebola viruses, respectively. Both have shown effectiveness against SARS-CoV-2 in clinical studies and have been repurposed for the treatment of COVID-19 patients in several countries. They are antimetabolites that mimic viral genome structural features and subsequently inhibit viral replication by targeting RNA polymerase [6–9]. FPV is usually prescribed orally for mild and moderate COVID-19 cases, whereas RDV is frequently administered parenterally for severe and hospitalized cases. A study on Syrian hamsters infected with COVID-19 discovered that co-administration of FVP and the RDV reduced the virus load in the hamsters’ lungs more effectively than either drug alone [10].

FPV was reported to be quantitatively determined alone by chromatographic [11–15], spectrofluorimetric [12, 16], spectrophotometric [15, 17], and electrochemical techniques [18–22], either in pharmaceutical dosage form or plasma. In addition, FPV was determined simultaneously in plasma with molnupiravir and ritonavir using the HPTLC technique [23], with hydroxychloroquine and RDV using the spectrofluorimetric method [24, 25], with dexamethasone and RDV using the UPLC technique [26], and with RDV using the TLC-densitometric [27] and spectrofluorimetric [28] techniques. On the other hand, different analytical approaches have been reported for the quantification of RDV alone in pharmaceutical dosage form or plasma, including chromatographic [29–34], spectrofluorometric [35, 36], and spectrophotometric methods [30, 37].

Regarding the pharmacokinetic profile of FPV and RDV, the C_max_ of FPV and RDV, 4.43 µg/mL and 3027 ng/mL, respectively, [24, 25, 38], are high enough to be detected spectrophotometrically in the plasma matrix. This inspired the authors to develop the first validated green spectrophotometric methods for the determination of FPV and RDV in plasma matrices. Due to the considerable overlap between the UV absorption spectra of FPV and RDV, which makes it challenging to directly resolve them in mixtures, we presented in this paper two ratio spectra manipulating spectrophotometric methods, ratio difference [39–41] and first derivative of ratio spectra [42, 43], for the measurement of FPV and RDV in pure forms and spiked plasma. Additionally, the greenness of the described methods was evaluated using three metrics systems, namely, the national environmental method index (NEMI) [44–46], the analytical eco-scale [47], and the analytical greenness metric (AGREE) [48–61].

## Experimental

### Materials and solvent

Pharmakeda Health Company (Egypt) provided FPV and RDV powders. Ethanol HPLC grade was supplied by Sigma-Aldrich (Germany). The National Egyptian Blood Bank provided a human plasma sample, which was kept frozen until analysis. Remdesivir-EVA PHARMA vial (100 mg RDV per 20 mL) and Avipiravir tablets (200 mg FPV per tablet) were obtained from local pharmacy.

### Instrument

All measurements were taken with a Shimadzu UV-Visible 1800 Spectrophotometer (Japan). The samples’ absorption spectra were scanned using 10 mm quartz cuvettes. The scanned spectra were manipulated using Shimadzu UV-Probe software version 2.43, accessed by Umm Al-Qura University, Makkah, Saudi Arabia.

### **Standard solutions**

FPV and RDV stock solutions with a concentration of 100 µg/mL were prepared by weighing 10 mg of each drug powder and placing it in two separate 100-mL volumetric flasks with 50 mL ethanol, vigorously shaking, and filling to the required volume with ethanol.

## Procedures

### Development of calibration graphs for analytical procedure

Separate serial dilutions of FPV and RDV were made by transferring aliquots of FPV and RDV equivalent to (20–240 µg) from their respective standard solutions (100 µg /mL) into two sets of 10-mL volumetric flasks and diluting to volume with ethanol. The absorption spectra of these dilutions were scanned and recorded against a blank in the wavelength range of 200–400 nm. The recorded zero-order absorption spectra of each drug were divided by a suitable divisor spectrum from the spectra of the second drug to create the ratio spectra. A spectrum of 10 µg/mL of each drug was the optimal divisor.

*Ratio difference method (RD).* The calibration graphs were created by plotting the difference in amplitude values of the ratio spectra at 222 and 256 nm for FPV and 247 and 271 nm for RDV versus the drugs concentrations, and the regression equations were generated.

*First derivative of ratio spectra method (*^*1*^*DD).* Each drug’s created ratio spectra were converted to its first order derivative using ∆λ = 4 and scaling factor 100. The ^1^DD amplitude values for FPV and RDV were measured at 228 and 251.20 nm, respectively. To obtain the calibration graphs, the recorded values were plotted against each drug concentration, and the corresponding regression equations were generated.

### Analysis of laboratory mixed solutions

Five laboratory mixed samples containing different concentrations of FPV and RDV in different ratios were made and analyzed as mentioned under the development of calibration graphs for analytical procedure.

### Analysis of pharmaceutical preparations

Five Avipiravir tablets with 200 mg FPV each were weighed and finely crushed. In a 100-mL volumetric flask with 60 mL ethanol, an exact weight of powder corresponding to one tablet was added. The flask was vigorously shaken for 20 min before being filtered and ethanol-adjusted to 100 mL. Five samples of varied concentrations were obtained after further dilution with ethanol, and the process outlined under the development of calibration graphs for analytical procedure was followed.

In contrast, the contents of three Remdesivir-EVA PHARM vials (100 mg RDV per 20 mL) were well mixed. An aliquot of 10 mg RDV was transferred to a 100-mL volumetric flask, properly mixed, and filled to volume with ethanol. The appropriate amounts of sample fraction were transferred into a series of 10-mL volumetric flasks, and the analytes quantities that fell within the calibration range were obtained by adding ethanol. The samples were analyzed using the procedure outlined under the development of calibration graphs for analytical procedure.

### Development of calibration graphs for bioanalytical procedure

Different spiked human plasma samples of FPV and RDV were made by transferring aliquots of FPV and RDV equivalent to (15–240 µg) and (14–240 µg), respectively, from their respective standard solutions (100 µg/mL) into two sets of centrifugation tubes together with 0.1 mL of human plasma and 5 mL of acetonitrile. The tubes were shaken for 1 min on a vortex mixer and then centrifuged for 30 min. The formed supernatants were evaporated in rotary evaporator to dryness. The residues were dissolved in appropriate volume of ethanol, transferred to 10-mL volumetric flasks, and diluting to volume with ethanol. Calibration curves were created as mentioned under Development of calibration graphs for analytical procedure. During the development of the calibration curve, 1.5 µg/mL and 1.4 µg/mL were taken as the lower limit of quantification (LLOQ) for FPV and RDV, respectively, and 24 µg/mL was taken as the upper limit of quantification.

Quality control samples were prepared at four different levels (LLOQ, middle quantifiable concentration “MQC” (100%, 13.00 µg/mL), lower quantifiable concentration “LQC” (80%, 10.40 µg/mL), and high quantifiable concentration “HQC” (120%, 15.60 µg/mL) and analyzed thereafter to evaluate accuracy and precision. The matrix effect is efficiently evaluated by analyzing LQC and HQC samples six times using a plasma matrix.

## Results and discussion

### **Spectral characteristic**

Analytical chemists have recently dedicated themselves to making analytical procedures more environmentally benign and safer for humans through using solvents and chemicals that have a low impact on the environment while lowering energy consumption and waste generation. In our study, we tried to follow the principles of green analytical chemistry by using a green solvents and energy-efficient apparatus while lowering waste generation. For such considerations, we conduct the study using a UV-Vis Spectrophotometer, which utilizes energy at a rate of less than 0.1 kWh per sample and produces little waste. Practically, FPV was found to be soluble in DMSO, ethanol, methanol, and water, while RDV was soluble in DMSO, ethanol, and methanol but insoluble in water. According to a survey of solvent selection guides [62], ethanol was selected as a solvent for the analysis of the studied drugs because it was ranked among the recommended solvents (green category).

**Fig. 2:**
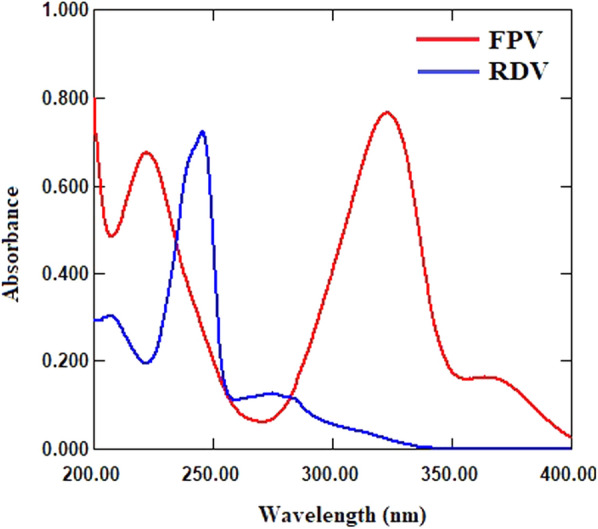
Absorption spectra of FPV (15 µg/mL) and RDV (15 µg/mL)

The normal UV absorption spectra of FPV and RDV overlap significantly, making their simultaneous determination extremely challenging (Fig. 2). Two ratio spectra manipulating spectrophotometric methods namely, ratio difference and first derivative of ratio spectra were used to overcome the overlap issue and quantitatively determine FPV and RDV in their laboratory mixed solutions and spiked plasma.

Theoretically, for mixture of two drugs (x) and (y), the absorption of two drugs at certain wavelength according to Beer’s law is

1 $${\text{A}}_{m} {\text{ = A}}_{x} {\text{ + A}}_{y} {\text{ = }}\varepsilon _{x} {\text{C}}_{x} {\text{ + }}\varepsilon _{x} {\text{C}}_{y}$$

where A_m_ is the sum of absorbance of two drugs A_x_ and A_y_; Ɛ_x_ and Ɛ_y_ are the molar absorptivity of two drugs at certain wavelength; C_x_ and C_y_ are the concentrations of two drugs.

For determination of drug (x) in mixture, the absorbance of drug (y) should be canceled by dividing the mixture by a known concentration of drug (y) as a divisor y° [A_y°_=Ɛ_y°_ C_y°_] as the following

2 $$\frac{{A_{m} }}{{A_{{{\text{y}} \circ }} }} = \frac{{A_{x} }}{{A_{{{\text{y}} \circ }} }} + \frac{{C_{y} }}{{C_{{{\text{y}} \circ }} }}$$

Simply the Eq. (2) can express as the following:

3 $${P}_{m}={P}_{x}+K$$

where K is a constant (C_y_/C_y°_); P_m_ is the ratio absorbance of the mixture to drug (y°); P_x_ is the ratio absorbance of the drug (x) to drug (y°).

The constant (K), along with any additional instrumental error or interference from the sample matrix, will be cancelled by selecting two wavelengths (λ_1_ and λ_2_) on the obtained ratio spectrum and subtracting the amplitudes at these two points, as the following

4 $$\varDelta P={P}_{m1}-{P}_{m2}= \left({P}_{x1} +K\right)-\left({P}_{x2}+ K\right)={P}_{x1} -{P}_{x2}$$

where ∆P is the difference between the peak amplitude at two selected wavelengths (λ_1_ and λ_2_); P_x1_ and P_x2_ are the peak amplitudes of the ratio spectra of the drug (x) at two wavelengths, respectively.

The drug (x) can be quantitively analyzed from a mixture by plotting the ratio spectra amplitude difference at two different wavelengths against the concentration of drug (x). Similarly, if drug (x) is present, another drug (y) can be determined using the same procedure.

**Fig. 3:**
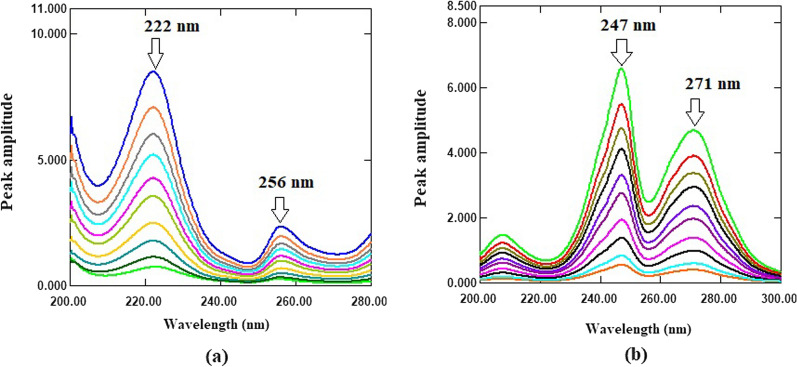
**a** Ratio spectra of FPV (2–24 µg/mL) using 10 µg/mL of RDV as a divisor; **b** Ratio spectra of RDV (2–24 µg/mL) using 10 µg/mL of FPV as a divisor

Accordingly, the ratio spectra of FPV and RDV were generated by dividing the normal drugs spectra by a suitable divisor spectrum (Fig. 3). Because selecting a divisor is such an important step with regards to signal-to-noise ratio and sensitivity, several FPV and RDV spectrums were tested as divisors. A spectrum of 10 µg/mL of each drug was found to be the best divisor. The ratio difference method is based on calculating the ratio spectra amplitude difference at two different wavelengths. As a result, the linearity at each wavelength must be verified. Linearity was found to be good at 222 and 256 nm for FPV and 247 and 271 nm for RDV. FPV was determined selectively in a mixture without interference from RDV using the equation derived from plotting the amplitude differences between 222 and 256 nm versus FPV concentrations. RDV, on the other hand, was determined without interference from FPV using the equation derived from plotting the amplitude differences between 247 and 271 nm versus RDV concentrations.

**Fig. 4:**
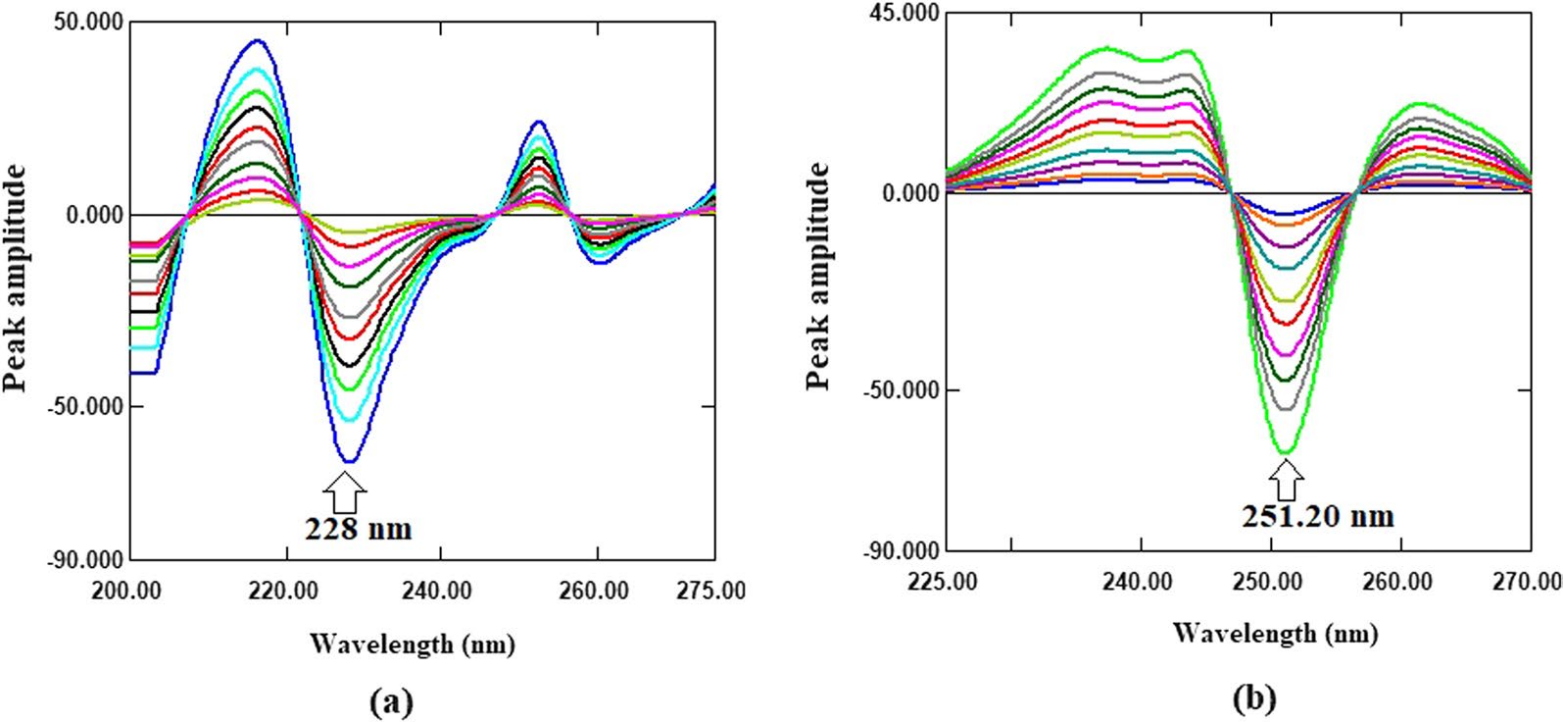
**a** ^1^DD spectra of FPV (2–24 µg/mL) using 10 µg/mL of RDV as a divisor; **b** ^1^DD spectra of RDV (2–24 µg/mL) using 10 µg/mL of FPV as a divisor

For first derivative of ratio spectra approach, the obtained ratio spectra of each drug were transformed to its first order derivative using ∆λ = 4 and scaling factor 100 (Fig. 4). The linearity and selectivity of the ^1^DD spectra of FPV and RDV at various peak amplitudes were investigated, and it was discovered that wavelengths of 228 and 251.20 nm for FPV and RDV, respectively, provided good linearity and selectivity. As a result, FPV was determined selectively in a mixture without interference from RDV using the equation derived from plotting the amplitude values at 228 nm versus FPV concentrations. RDV, on the other hand, was determined selectively in a mixture without interference from FPV using the equation derived from plotting the amplitude values at 251.20 nm versus RDV concentrations.

## Method validation

### Validation of Analytical Procedures

The procedures used were validated in accordance with ICH Q2 (R1) recommendations [63]. The data in Table 1 indicate the limits of detection (LOD) & quantitation (LOQ), accuracy, precision, linearity, and regression parameters. The methods used indicated satisfactory linearity in the concentration ranges of 2–24 µg/mL for both FVP and RDV, with acceptable accuracy and precision. The methods were discovered to be sensitive, with LOD values of 0.390 µg/mL and 0.336 µg/mL for FPV and RDV, respectively, in the ratio difference method, and 0.402 µg/mL and 0.331 µg/mL for FPV and RDV, respectively, in the first derivative of ratio spectra method. The described procedures demonstrated successful applicability in terms of selectivity for measurement of FPV and RDV in their laboratory mixed solutions without interference from each other (Table 2), as well as in pharmaceutical dosage forms (Table 3), without interference from excipients, as confirmed by standard addition technique results (Table 3). The obtained results were statistically compared to the reported methods [17, 37] using the student’s t-test and F-test. The results found no statistically significant difference between the methods (Table 3).

**Table 1:** Regression and validation data for quantitative analysis of FPV and RDV by the proposed methods

Parameters	RD		^1^DD	
	FPV	RDV	FPV	RDV
Wavelength (nm)	222 & 256	247 & 271	228	251.2
Linearity range (µg/mL)	2–24	2–24	2–24	2–24
Slope	0.2571	0.0784	2.7023	2.7526
Intercept	− 0.0023	− 0.0004	− 0.1100	− 0.0123
Coefficient of determination (r^2^)	0.9997	0.9998	0.9997	0.9998
LOD (µg/mL)	0.390	0.336	0.402	0.331
LOQ (µg/mL)	1.182	1.018	1.217	1.002
Accuracy (%R)^a^	100.32	99.89	100.46	99.67
Repeatability precision (RSD)^b^	0.555	0.695	0.492	0.672
Intermediate precision (RSD)^b^	0.701	0.815	0.716	0.850

^a^ Average of 9 determinations (3 concentrations repeated 3 times)

^b^ RSD of 9 determinations (3 concentrations repeated 3 times)

**Table 2:** Application of the proposed methods for the determnation of FPV and RDV in their laboratory mixed solutions

Added (µg/mL)		%Recovery			
		RD		^1^DD	
FPV	RDV	FPV	RDV	FPV	RDV
5	5	100.76	99.34	101.18	99.65
10	5	99.89	99.59	99.54	99.01
5	10	99.60	100.82	100.29	99.62
10	10	100.40	99.67	100.77	100.56
20	10	100.22	100.94	99.83	100.89
Mean ± RSD		100.17 ± 0.450	100.07 ± 0.749	100.32 ± 0.665	99.95 ± 0.762

**Table 3:** Quantitative determination of FPV and RDV in pharmaceutical preparations using the proposed methods, as well as the results of using the standard addition technique, with statistical comparison to the reported methods

Recovery ± RSD							
FPV				RDV			
Method	RD	^1^DD	Reported method [17]	Method	RD	^1^DD	Reported method [37]
Avipiravir tablets ^a^	99.46 ± 0.765	100.23± 1.099	100.03 ± 1.169	Remdesivir- vial ^a^	99.51 ± 0.658	100.20 ± 1.040	100.14 ± 1.319
Standard addition ^b^	100.55 ± 0.986	100.12 ± 0.887		Standard addition ^b^	100.98 ± 1.058	100.42 ± 0.974	
*t*-Test (2.306)^c^	0.905	0.869		*t*-Test (2.306)^c^	0.120	0.085	
*F*-test (6.388)^c^	2.364	3.191		*F*-test (6.388)^c^	1.440	1.607	

^a^Average of five determinations

^b^Average of three determinations

^c^ The values in parentheses are tabulated values of *t* and *F* at P = 0.05

### Validation of Bioanalytical Procedures

The validation of the developed methods was done as per ICH M10 guidelines [64], including linearity, the lower limit of quantification (LLOQ), accuracy, precision, and matrix effect. Linearity was evaluated at seven calibration standard levels. The linearity ranges for FVP and RDV were 1.5–24 g/mL and 1.4–24 g/mL, respectively, with a coefficient of determination (r2) ≥ 0.9971 (Table 4). The LLOQ is the lowest amount of FVP and RDV that can be quantitatively determined and calculated with acceptable precision and accuracy. As listed in Table 4, the LLOQs of FVP and RDV were 1.5 µg/mL and 1.4 µg/mL, respectively.

The accuracy of the described methods was performed based on by calculating the mean percentage recovery of FVP and RDV from four quality control samples including LLOQ, middle quantifiable concentration (MQC) (100%, 13.00 µg/mL), lower quantifiable concentration (LQC) (80%, 10.40 µg/mL), and high quantifiable concentration (HQC) (120%, 15.60 µg/mL). Triple measurement of the concentrations was done using the calibration graph of the spiked plasma sample and the mean percent recovery was calculated. The calculated percentage recovery of FVP and RDV in the spiked plasma was found to be greater than 95% demonstrating that the described method was highly accurate (Table 4).

Precision of the described methods was studied through calculating the percent of relative standard deviation for triplicate determination of LLOQ, LQC, MQC and HQC values for every drug within one day for repeatability and on three successive days for Inter mediate precision. The small values of %RSD demonstrated high precision of the proposed models as listed in Table 4.

Matrix effect is defined as the change in the determined compound response due to interfering components in the sample matrix. So, matrix effect is efficiently evaluated by analyzing LQC and HQC samples six times using plasma matrix. The accuracy and precision should be in between ± 15%. The percentage recovery and %RSD was calculated. The assessed percentage recovery was found to be above 95% for all samples indicating noninterference of any unidentified compound in the sample matrix. Furthermore, the %RSD was less than 2. which confirmed absence of a plasma matrix effect on the drugs under the study and subsequent applicability to be used in bioanalysis as listed in Table 4.

**Table 4:** Bioanalytical validation parameters for quantitative analysis of FPV and RDV by the proposed methods

Parameters	RD		^1^DD	
	FPV	RDV	FPV	RDV
LLOQ (µg/mL)	1.5	1.4	1.5	1.4
Linearity range (µg/mL)	1.5–24	1.4–24	1.5–24	1.4–24
Accuracy (%R)^a^	95.74	96.53	96.89	95.02
Repeatability precision (RSD)^b^	1.567	1.897	1.418	1.658
Intermediate precision (RSD)^b^	1.774	2.001	1.704	1.911
Matrix effect (Mean ± RSD)	96.30 ± 1.720	95.14 ± 1.936	96.25 ± 1.340	95.81 ± 1.437

^a^ The mean percentage recovery of tripicate determination of four quality control samples

^b^ RSD of tripicate determination of four quality control samples

## Greenness evaluation

The evaluation of the described spectrophotometric methods in the context of green analytical chemistry was performed using three greenness assessment metrics, namely the national environmental method index (NEMI), the analytical eco-scale, and the analytical greenness metric (AGREE). Additionally, a comparison of the described methods greenness to the reported UPLC method was carried out.

The national environmental method index (NEMI) is the first reported qualitative greenness assessment tool. It based on a simple pictogram of four quadrants represents four criteria (amount of waste generated, reagents that are persistent, bioaccumulative, or toxic (PBT), consumption of hazardous, whether the conditions are corrosive). These criteria are evaluated in a binary manner: if a criterion’s value is satisfied, the corresponding section of the pictogram is filled in with green colour; otherwise, it stays uncolored. The described spectrophotometric methods met the four criteria and were shaded green in the four quadrants of the pictogram because ethanol was not reported as a PBT or hazardous reagent, the procedures were not performed in corrosive pH media, and the amount of waste generated was less than 50 g/sample (Table 5). In contrast, the reported UPLC method had three out of four quadrants colored green due to the presence of hazardous solvents in the mobile phase. As a result, the spectrophotometric approaches presented here are greener than the reported UPLC method.

The analytical eco-scale is a semi quantitative metric built on the concept that ideal green analysis has a value of 100. Penalty points (PPs) are assigned for each analytical procedure parameters (amount of reagents, hazards, energy and waste) deviated from ideal green analysis. The penalty points of whole procedure parameters are summed and subtracted from the basis of 100. More than 75 score is excellent green analysis, less than 50 score is inadequate green analysis, and between 50 and 75 score is acceptable green analysis. The calculated analytical eco-score of the described spectrophotometric methods (93 score) versus the reported UPLC method (83 score) supported the NEMI results and favored the spectrophotometric method, which uses just ethanol as a solvent and has no occupational hazards (Table 5).

The analytical greenness measure (AGREE) is a user-friendly software that uses the twelve-significance principle of green analytical chemistry as input criterion. Each of the twelve inputs is scored on a common scale ranging from 0 to 1 and mirrored on the intuitive red-yellow-green colour scale. Furthermore, the weight of each input criterion is assigned according to its importance in the procedure, and this is reflected by the width of its corresponding segment. The output is a clock-like graph, with the overall score and color representation in the middle. The perfect analysis has a score of one and is represented by a dark green color. The AGREE pictogram in Table 5 displayed the ultimate score (0.75) of the described spectrophotometric methods, indicating excellent green analysis.

**Table 5:** Greenness evaluation and comparison of the developed methods and reported one

	Developed spectrophotometric methods		Reported UPLC method [26]	
NEMI				
Analytical Eco-scale	**Parameters**	**PPs**	**Parameters**	**PPs**
	***Reagents***		***Reagents***	
	Ethanol	4	Methanol	6
			Acetonitrile	4
			Water	0
			Orthophosphoric acid	1
	***Instrument spectrophotometer***		***Instrument UPLC***	
	Energy: <0.1kWh per sample	0	Energy: <0.1kWh per sample	0
	Occupational hazards	0	Occupational hazards	3
	Waste < 10 mL	3	Waste (1–10 mL)	3
	***Total PPs***	**7**	***Total PPs***	**17**
	***Analytical Eco-scale score***	**93**	***Analytical Eco-scale score***	**83**
AGREE tool				

## Conclusion

In this work, we developed two green UV spectrophotometric methods; ratio difference and first derivative of ratio spectra methods for the analysis of FPV and RDV in spiked plasma. Overall, the two methods were found to be simple, accurate and do not require sophisticated techniques or instruments. They are also sensitive and selective, making them suitable for routine analysis of FPV and RDV in plasma. The ratio difference method is simpler than the first derivative of ratio spectra method because it does not require critical measurements at fixed wavelengths or any derivative calculation. The adherence of the developed method to the principles of green analytical chemistry was evaluated using NEMI, the analytical eco-scale, and AGREE tool. The developed methods showed a closely adherence to the principles of green analytical chemistry when compared to the reported UPLC method, which utilized hazardous reagents in the mobile phase and generated more waste.

## Data Availability

The datasets used and/or analyzed during the current study available from the corresponding author on reasonable request.

## Abbreviations

FPV: Favipiravir
RDV: Remdesivir
RD: Ratio difference method
^1^DD: First derivative of ratio spectra method
NEMI: National Environmental Method Index.
